# Editorial: Immunotherapy resistance and advancing adaptive cell therapeutics

**DOI:** 10.3389/fimmu.2025.1640317

**Published:** 2025-06-24

**Authors:** Luciana Rodrigues Carvalho Barros, Amar Yeware

**Affiliations:** ^1^ Faculdade de Medicina FMUSP, Universidade de São Paulo, São Paulo, SP, Brazil; ^2^ Wisconsin Institutes for Medical Research, University of Wisconsin–Madison, Madison, WI, United States

**Keywords:** oncoimmunology, adaptive cell therapy, chimeric antigen receptor (CAR), immunotherapy, resistance

Advances in the understanding of immune biology and the development of a newer generation of immunotherapies have ushered in a new stage in combating many conditions, including cancer, autoimmune diseases, and infectious diseases. The principal focus of this type of therapy is to modulate the host immune response using antibodies, vaccines, cytokines, and cells. However, it is important to note that the immune responses are dynamic and constantly developing. The response to immunotherapy varies among patients and includes their pathophysiological environment, metabolism, and genetic factors. Additionally, Advanced Cell Therapeutics (ACT) is becoming more prevalent in the treatment of all kinds of diseases; still, a substantial number of patients are experiencing resistance to many of these treatments, necessitating further advancements. Therefore, researchers are pursuing different modifications to ongoing therapeutic options to overcome immunotherapy resistance and emphasize the promising frontier of adaptive cell therapeutics in overcoming these challenges.

## Chimeric antigen receptor cell therapies

CAR-T cells have been used with considerable success to treat hematologic cancers. However, manufacturing autologous CAR-T cells is challenging because of the quantity and quality of the patient’s T cells, which can compromise the clinically applicable dose of CAR-T cells, increase the risk of relapse during production, and cause manufacturing difficulties. One approach to overcoming these challenges is an “off-the-shelf” production derived from allogeneic T cells from peripheral blood (PB), embryonic or iPSC-derived cells, and umbilical cord blood (UCB). Rassek et al. compared the autologous T cells with these two allogeneic sources in production time, cost, quality of cells, availability, quality control, applicability, T cell exhaustion, and graft-versus-host disease risk. There is preclinical and clinical evidence from phase I trials for UCB-derived CAR cells. One of the advantages of using UCB-derived CAR cells is the abundance of cells in cord blood banks, which makes it possible to obtain young, naive natural killer (NK) cells, T cells, and other types of cells, including mesenchymal stem cells (MSCs). Although cord blood needs *in vitro* purification and expansion, it effectively has a lower level of checkpoint inhibitors, such as PD1, LAG3, and TIM3 expression, than allogeneic PB-derived CAR cells. 

Regardless of the cell source, T cells and NK cells are the main effector cells carrying CARs for cancer therapy. A main concern with CAR-T or CAR-NK cells is their ability to migrate and persist in the tumor. All immune cells migrate via a chemokine gradient into the tumor microenvironment. In the case of myeloma, the bone marrow is the main site, and the chemokine CXCL12 may attract NK and T cells, thereby increasing the likelihood of therapy success. Moles et al. developed a BCMA CD28 zeta CAR-NK cell with bicistronic CXCR4 or CXCR4^R334X^ surface receptor expression. Both receptors increase the *in vitro* migration and cytotoxicity of CAR-NK cells against RAJI^BC eMA^, and trogocytosis. CXCR4 expression also identifies a lower amount of antigen that is necessary for BCMA-CAR activation, evidencing a potential recognition of BCMA^low^ cells.

Another interesting strategy to increase CAR potential is changing the scFv (single chain Fragment variant) to a Variable Heavy domain of a Heavy chain (VHH) molecule. Hanssens et al. tested a library of VHH-CARs derived from camelid-found heavy-chain-only antibodies (HCAbs) as an antigen-binding moiety against the CS1 antigen for multiple myeloma. Several VHH-CAR T cells could be activated *in vitro*, exhibited cytotoxicity, and were able to migrate to the tumor *in vivo*. Nonetheless, *in vitro* predictions failed to indicate the best VHH behavior.

For solid tumors, ACT is challenging due to low cell migration, an inhibitory microenvironment, and especially antigen expression heterogeneity within the tumor. CAR-T cells, NK cells, dendritic cell-based vaccines, and tumor-infiltrating lymphocytes (TILs) are the main strategies presented for the treatment of solid tumors. Several studies were reviewed by Ao et al. on biliary tract malignancies, intrahepatic cholangiocarcinoma, extrahepatic cholangiocarcinoma, and gallbladder cancer. These tumors are aggressive, with a poor prognosis and an overall survival rate of only a few months despite chemotherapy. These tumors have a low incidence in Western countries, but a 40-fold higher incidence in Asian countries. A combination of ACT may be tested in the future as an alternative to the current chemotherapy to improve the prognosis.

In ACT, especially when expanding and re-injecting TILs or CAR-T cells, the functional quality of these cells could become critical. For example, the meta-analysis by Wan et al. indicated that high PD-1 expression on CD8+ cells based on 20 studies involving 3,086 patients, was linked to poorer overall survival (Yan et al.). However, if the injected T cells have already acquired an exhausted phenotype such as PD-1 upregulation in the tumor microenvironment, their anti-tumor efficacy may be compromised. Furthermore, the study suggested that the use of checkpoint inhibitors such as Pembrolizumab in combination with chemotherapy could lead to cytokine release syndrome or hemophagocytic lymphohistiocytosis (Qin et al.). Therefore, strategies that either select for non-exhausted adaptive T cells or genetically knockout T cells for checkpoints are needed to enhance ACT outcomes.

Adaptive cell and CAR cell-based therapies are currently in clinical use for cancer, especially hematological cancers, but face challenges in terms of availability and cost, which limit their use for more patients. On the other hand, adaptive cell therapies for autoimmune diseases are in Phase 1 or 2 clinical trials. Fu et al. found that autoimmune diseases such as systemic lupus erythematosus, rheumatoid arthritis, and multiple sclerosis can be managed by pharmacotherapy or ACT. In the latter, regulatory T cells (Tregs), CAR-Treg cells, chimeric auto-antibody receptor T cells, regulatory NK cells, and tolerogenic dendritic cells can be used as ACT. Although none of these strategies are approved for autoimmune diseases, a combination of drugs and ACT seems promising for the future.Various approaches are being investigated to enhance ACT and immunotherapeutics, including using machine learning to integrate multi-omics data for precise prognostic modeling. Yan et al. identified an immunogenic cell death-related signature (ICDRS) using single-cell and bulk RNA sequencing data, offering valuable insights into tumor immune evasion in bladder cancer. This approach could make it possible to identify patient-specific features, enabling more personalized ACT or its combination with immunotherapeutic strategies.

In conclusion, ACT and advanced immunotherapeutics hold immense potential for treating cancer and autoimmune diseases by developing and engineering immune cells in different ways alongside CAR receptors. However, current challenges such as resistance, cell exhaustion, and manufacturing hurdles persist; ongoing innovations and personalized approaches could pave the way for more effective and accessible therapies.

